# The assessment of an extended set of socio-economic determinants to explain anxiety and uncertainty, insufficient quality and food intake of Afghan refugees

**DOI:** 10.1017/S1368980021004043

**Published:** 2022-03

**Authors:** Mohammad Reza Pakravan-Charvadeh, Hassan Vatanparast, Edward A Frongillo, Mahasti Khakpour, Cornelia Flora

**Affiliations:** 1 Department of Agricultural Economics and Rural Development, Faculty of Agriculture, Lorestan University, 5th Kilometer of Khorramabad-Boroujerd Highway, Khorramabad 6815144316, Lorestan, Iran; 2 College of Pharmacy and Nutrition, University of Saskatchewan, Saskatoon, Saskatchewan, Canada; 3 Health Promotion, Education, and Behavior, Arnold School of Public Health, South Carolina, USA; 4 Saint Francis Xavier University, Antigonish, Nova Scotia, Canada; 5 Department of Sociology, Iowa State University, Iowa, USA

**Keywords:** Afghan refugees, Associated factors, Food insecurity, Quantitative method, Urban areas

## Abstract

**Objectives:**

In this study, socio-economic factors associated with Afghan refugee households’ food insecurity, anxiety and uncertainty, insufficient quality and food intake were determined.

**Design:**

Household Food Insecurity Assess Scale measurement was applied to assess food insecurity, anxiety and uncertainty, insufficient quality and insufficient food intake. Descriptive analysis and multivariable regression models were used to determine the associated factors.

**Setting:**

The study was carried out in urban areas of Tehran province in Iran.

**Participants:**

To collect data, interviews were conducted among 317 Afghan households. The questionnaire was administered via face-to-face interviews to either the breadwinner of the selected households or a member who could respond on behalf of the household.

**Results:**

About 11·3 % of Afghan households who resettled in Tehran province were food secure, while 11·7 % were marginally, 40·7 % moderately and 36·3 % severely food insecure. Economic and financial factors were inversely and significantly associated with food insecurity. Employment, income, distance from the central market and personal saving were inversely associated with food insecurity, while other determinants, including the length of living time in Tehran, house type and the number of male and female children, had a direct association with food insecurity.

**Conclusions:**

The associations of socio-economic factors with three categories of food insecurity differed. Elimination of occupation bans that the Iranian government imposes on refugees provides simple access to financial supports like long-term loans, and opening a bank account for refugees will benefit both Iranians and refugees.

Food insecurity is defined as ‘the inability to access sufficient, safe, and nutritious food to maintain a healthy and active life’. Notwithstanding the growth in global wealth, many populations are vulnerable to food availability and accessibility^(1–3)^. Refugees who resettle in new areas or countries, especially in developing countries, are particularly vulnerable to food insecurity^(4,5)^. The term ‘refugee’ refers to ‘a person who is unable or unwilling to return to their country of nationality because of persecution or a well-founded fear of persecution on account of race, religion, nationality, political opinions, or membership in a particular group’^(6)^. About sixty million people have to flee from expulsions or civil wars or abandon their country to escape from poverty worldwide^(7)^. Displacement from their usual environment makes refugees vulnerable to poor quality of life and mental health^(8,9)^.

One of the largest refugee groups in the world is from Afghanistan^(6)^. During the 1978 Saur Revolution in Kabul, the Russian invasion in 1979 and the US invasion in 2001, Afghan refugees moved to adjacent countries, particularly Iran and Pakistan, to escape the war. There were about 2·7 million Afghan refugees, at the end of 2018, mainly due to births during the year compared with 2·6 million a year earlier. In 2019, about 1 403 500 Afghan refugees were living in Pakistan as the largest host country^(6)^. About 951 100 Afghan refugees fled to the Islamic Republic of Iran at this time. According to some reports, there are nearly one million illegal and undocumented refugees in Iran^(6)^. Iran has 46 % of all Afghan refugees in Asia, 38 % of all Afghan asylum seekers around the world (among fifty-six countries) and almost 56 % of all illegal Afghan refugees. Given the prevalence of war and the history of displacement in Afghanistan, many human rights reports and academic studies focused on Afghan refugee health status^(10)^. Others have evaluated food in/security status beyond the borders of Afghanistan and its associated obstacles^(11–19)^.

To our knowledge, this is the first study that determines the associated factors with different dimensions of the food security status of Afghan refugees in Iran. Determining associated factors will allow local and international non-governmental organisations to improve planning for improving refugees’ food security status^(20)^, make it easier for them to cope with post-resettlement obstacles and help them control post-traumatic stress disorders^(21)^.

Extant studies illustrated that the factors determining food security are complex and interrelated, encompassing political, socio-economic and environmental issues from poverty and inequality to social rights protection, and health, to name but a few^(1,22)^. The results of these studies revealed that some factors have an inverse association with refugees’ food insecurity in a specified study location, while the same factors have a direct association with refugee food insecurity in other locations at the same time. Although the goal of this study is analogous to other studies on refugees’ food insecurity around the world, it is innovative in determining factors associated with the food insecurity of Afghan refugees residing in Iran. The most innovative part of the study is the opportunity to test how three sub-scores, anxiety and uncertainties, insufficient quality and insufficient food intake, are associated with the individual and household characteristics. The lack of the study assessing refugees’ food security may be related to several difficulties in sampling, such as scattered refugees across the country as illegal and undocumented people and the resulting sense of insecurity of Afghan refugees in participating in detailed research that might result in deportation. By using a standard survey to assess food insecurity among Afghan refugee households in urban areas of Tehran province in Iran, four research hypotheses will be addressed in this study as follows:

Hypothesis 1: Most of the Afghan refugee households face food insecurity in the target area.

Hypothesis 2: Households’ characteristics are associated with Afghan refugees’ food security.

Hypothesis 3: Economic factors are indirectly associated with the anxiety and uncertainties of Afghan refugees.

Hypothesis 4: The associations of socio-economic factors with three categories of food insecurity differ.

## Methods

### Cross-sectional design

**Fig. 1:**
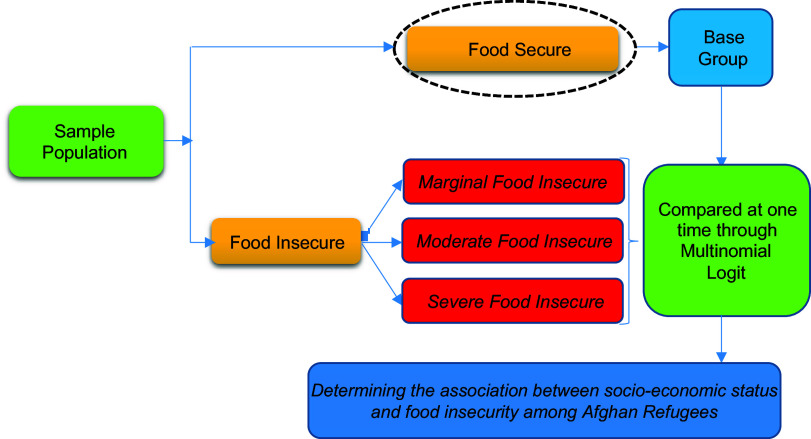
Cross-sectional design of the study

A cross-sectional analysis was used to follow our hypotheses (see Fig. 1). In the first stage, the data and information were gathered through a detailed questionnaire. Then, the food insecurity of Afghan refugees’ households was calculated and collapsed into three categories. Then the association of socio-economic factors with Afghan refugees’ categories of food insecurity was calculated through a multivariable regression model.

### Study area

**Fig. 2:**
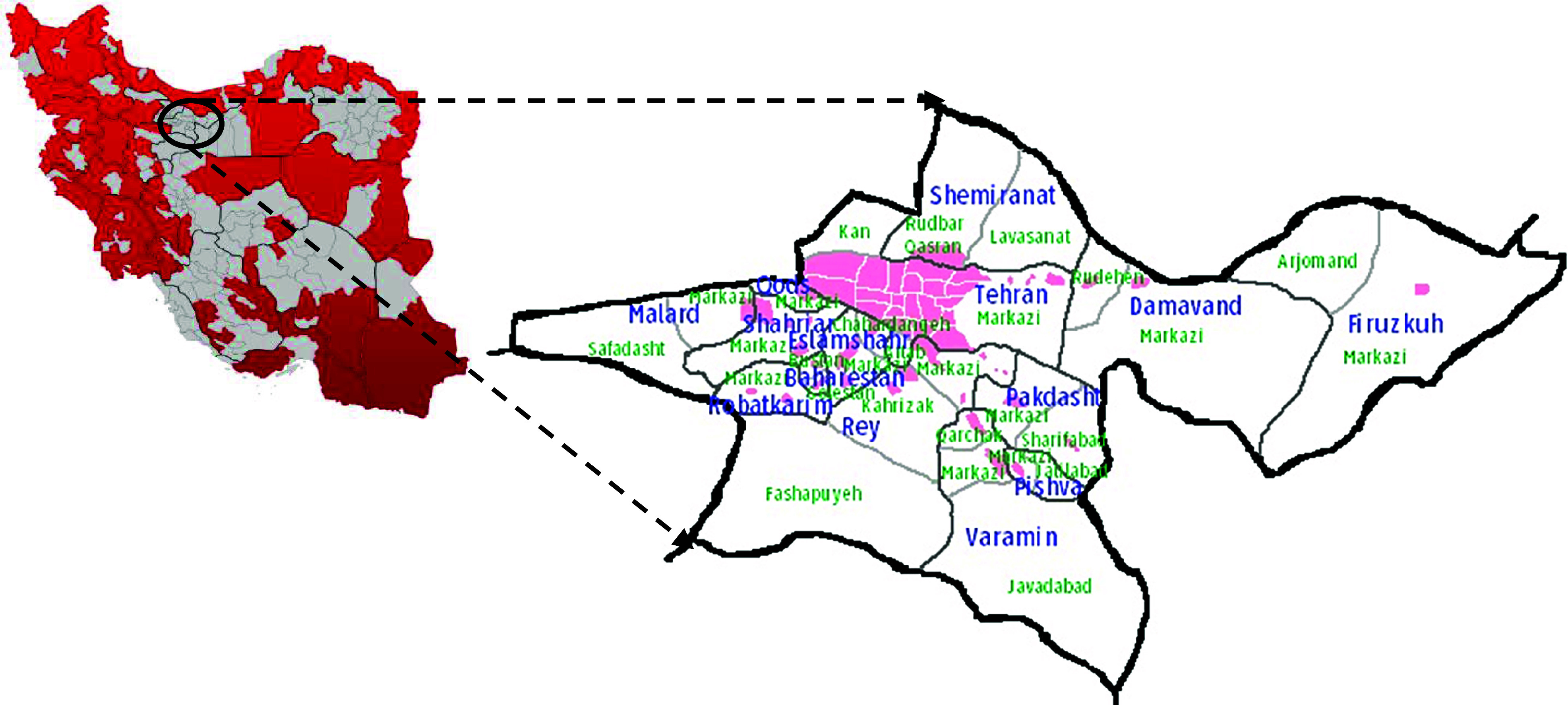
Map of the location of the studied area in the north of Iran, Tehran province

Data were collected in urban areas of Tehran province, the most densely populated province in Iran and the second-largest metropolitan area in the Middle East. According to the Iranian Statistical Center, 515 567 Afghan refugees live in this province, a figure that comprises almost 25 % of all the Afghan refugees residing in Iran. Afghan refugees have been prohibited from living in fifteen provinces (Fig. 2, marked in red) and are only able to live in specific regions and counties of twelve provinces^(23)^. They can live in three provinces without restriction: Tehran, Alborz and Qom.

### Study population

We used a two-staged cluster sampling. At first, cities (Shahrestan) were randomly selected within the province of Tehran, and at the second stage, households were selected randomly. We selected five cities, including Shahr-e-Rey, Shahriar, Robat Karim, Eslamshahr and Varamin. Then, we used the official documents of the Afghan refugees in the Iranian health centre in each city to select random sampling. (We considered the number of their recorded documents for arranging and selecting random sampling.) The questionnaire was administered via face-to-face interviews with either the breadwinner of the selected households or a member who could respond on behalf of the household.

### Data collection

The study involved a cross-sectional analysis of 317 Afghan refugee families residing in targeted areas of Tehran province. To conduct the interviews, we recruited fifteen Iranian and Afghan interviewers. All interviewers were trained in a session of about 6 h. They learned how to communicate well with Afghan refugees and how to complete the questionnaire. The language of Afghan and Iranian interviewers was Persian. Then they conducted in-person interviews with people from the households. The interviews were completed during a 2-months period (December 2018–January 2019). Informed consent was obtained from each respondent. We described the benefits of this study for respondents as making known the problem of Afghan refugees to governmental administrations and global organisations and contributing to encouraging policymakers to enact laws supporting Afghan refugees in Iran^(23)^.

### Assessment of food insecurity

**Fig. 3:**
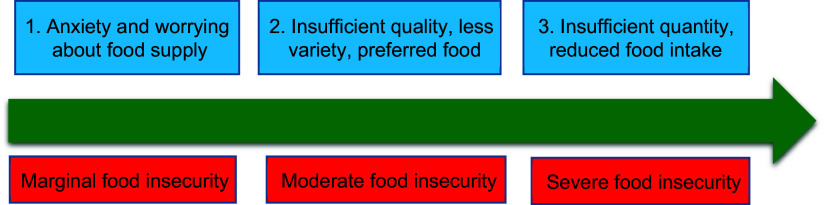
The domains of food insecurity among refugees

Food insecurity was assessed through a modified version of the Household Food Insecurity Access Scale (HFIAS) as a standard tool that has been validated in Iran^(24)^. The tool is a nine-item scale, with a reference period of the past 4 weeks for questions included^(24,25)^. Using HFIAS, households are asked to respond to each experience as never, rarely, sometimes or often, generating a total score from 0 to 27^(25)^. A higher score indicates greater household food insecurity. In the HFIAS scale, food insecurity is collapsed into three categories (i.e. marginal, moderate and severe) that correspond to uncertainty about the food supply, insufficient quality of food and insufficient quantity of food (Fig. 3).

We found that the representative of the breadwinner of slightly food-insecure households worries about future access to food or food affordability^(4)^. Moderate indicates that households lack the means to purchase foods with higher nutritional quality. Severe indicates food shortages and hunger, as adults and children in the household skip meals and/or cut portion sizes due to lack of resources available to purchase needed food ingredients^(4,26)^. The state of anxiety and uncertainty of Afghan households was calculated by question 1, insufficient quality was assessed by questions 2–4 and insufficient food intake was surveyed via items 5–9 (Table 1).

**Table 1:** The brief results of HFIAS questionnaire

	Question	Never	Rarely	Sometimes	Often	Mean	SD	Skewness	Kurtosis
Anxiety and uncertainty	Q1) Worry about food	38·2	28·4	22·7	10·7	1·05	1·01	0·49	1·03
Insufficient quality	Q2) Unable to eat preferred foods	29	35	21·7	14·3	1·21	1·01	0·38	1·03
	Q3) Eat a limited variety of foods	25·9	32·2	24·9	17	1·33	1·04	0·21	1·87
	Q4) Eat foods that you really did not want to eat	28·4	31·2	27·1	13·3	1·25	1·01	0·24	1·93
Insufficient food intake	Q5) Eat a smaller meal	39·1	37·6	16·7	6·6	0·90	.090	0·72	1·66
	Q6) Eat fewer meals in a day	52·7	32·5	10·1	4·7	0·66	0·84	1·16	1·64
	Q7) No food to eat of any kind in the household	69·4	19·9	9·1	1·6	0·43	0·72	1·60	1·72
	Q8) Go to sleep at night hungry	79·8	14·8	3·8	1·6	0·27	0·60	1·75	1·80
	Q9) Go a whole day and night without eating	88·3	11	0	0·63	0·13	0·38	1·77	1·73

Reference: authors’ results. HFIAS, Household Food Insecurity Assess Scale.

### Socio-economic factors

Potential control variables, such as the length of living time in Iran, the frequency of travel to Afghanistan, health and medical conditions of households’ members along with their properties, were added to the questionnaire. We then circulated it to 154 prominent scholars in health economics and public health across the world, including USA, Canada, Australia and Iran, and received suggestions to improve the questionnaire. All factors used in the final questionnaire are demonstrated in Appendix (Table A1). The descriptive statistics were reported as maximum, minimum, mean, SD, skewness and kurtosis for all factors. Finally, the socio-economic factors associated with different categories of food insecurity were examined by using a multivariable regression model.

### The model specification

After determining factors which were used in the final multivariable regression model and calculating HFIAS, the variance inflation factors were examined to test multi-collinearity between independent factors^(27,28)^. The analysis was conducted using the original score. In the first step, factors associated with a total score of food insecurity on Afghan refugee households were determined. Then, three categories of food insecurity – anxiety and uncertainties (original score between 1 and 3), insufficient quality (original score between 1 and 9) and insufficient food intake (original score between 1–15) – were considered as dependent variables in separate multivariable regression models. These relations were regressed upon an entire set of determinants hypothesised to have an association with Afghan households’ food insecurity, anxiety and uncertainties, insufficient quality and insufficient food intake^(29)^. The associated factors were extracted from the literature and the related papers. To choose the final factors in the regression models, correlation tests between each factor and dependent variable was carried out^(30)^.

## Results

### Demographic characteristics

Most of the breadwinners (head of household) were male (93 %), the average age of the head of the household was 41·5 years and the average size of households was 4·2 person (Table 2). Over half of the respondents (65 %) had a permanent job, 14 % had a seasonal occupation and 21 % was unemployed. Forty-four percent of Afghan households earned a monthly salary between 10 and 35 million Rials (between $45–$160), and only 6 % of the total sample earned a herd of money (More than 35 Million Rial ($160) per month). Afghan households had lived an average of 19 years in the study location. Most Afghan refugees (68·8 %) rented their home, while only 13·9 % of respondents reported owning their home. In our sample, the average amount of Rahn (the amount of deposited money in refundable-deposit-lease-contract, million Rial/year), as a guaranty to ensure the renter will give back home in good condition and pay the monthly rent on time. After finishing the time of contract, he/she will retake this deposited money. This type of contract for renting a home is known ‘Rahn’ in Iran and was 136 million Rials ($618). The average size of the respondents’ house was 74·1 m^2^, the average distance of Afghan households from the central market was 8·5 km and the frequency of travel to Afghanistan was 1·05 times during their resettlement. One-hundred twelve Afghan households had at least one child under the age of 5 years, 193 households had at least one child between the age of 5 and 18 years, and 123 had at least one child more than 18 years in the home. They had, on average, one boy and one girl and at least one student in their households. Of the breadwinners, 234 (73·8 %) were smokers. Of the mothers, 92·4 % were unemployed and had an average age of 35·7 years; only 15 % of mothers were engaged in handicraft activity. Most respondents (75 %) reported no personal savings. Before migrating to Iran, 25 %, 15 % and 15 % of the respondents lived in Daykundi, Kabul and Herat provinces, respectively, while the remaining 45 % of respondents lived in different locations in Afghanistan such as Mazar-i-Sharif, Kandahar, Bamyan, Balkh, Helmand, Badakhshan, Samangan and Ghazni cities. A majority were employed in shopkeeping or transportation as permanent or seasonal jobs.

**Table 2:** Descriptive analysis of independent variables

Factors	Min	Max	Mean	SD	Skewness	Kurtosis
Food security score	1	27	7·37	5·30	0·83	0·29
Age of HH	17	80	41·46	13·43	0·41	−0·42
Education level of HH	1	4	2·48	1·50	0·07	2·00
Employment status of HH	1	3	1·55	0·82	0·97	0·80
Income level	1	4	1·72	0·82	1·56	1·80
Length of stay in Iran	1	55	19·54	11·85	0·24	−0·41
Rent amount (million Rial)	0	1·8	0·14	0·19	1·67	1·52
Rahn (million Rial)	0	900	136·82	15·02	1·26	2·00
House size	0	250	73·91	39·37	1·04	1·36
Distance to city centre	0	65	8·44	9·72	1·09	1·37
Frequency of travel to Afghanistan	0	25	1·05	2·69	1·34	1·56
The number of male children	0	7	1·34	1·23	0·91	1·11
The number of female children	0	5	1·11	1·12	0·92	0·44
The number of students	0	5	1·04	1·15	0·87	−0·01
The number of employed members	0	5	0·59	1·06	1·14	1·67
The number of disease members	0	3	0·14	0·48	1·79	1·82
The number of illiterate members	0	6	0·84	1·11	1·63	1·92
The number of elementary members	0	5	0·99	1·09	0·95	0·27
The number of high school members	0	5	0·50	0·89	1·15	1·57
The number of university members	0	5	0·16	0·58	1·63	1·38
Age of mother	17	80	35·12	13·70	−0·24	0·83
Smoking status of HH	0	30	10·2	2·44	1·08	−0·82
Personal saving in banks	1	4	1·24	0·43	1·18	−0·60

Rial is the Iranian currency. HH, Head of Household.

### Food insecurity

Ten percent of interviewed Afghan households worried that they would not have enough food in the next 4 weeks. Thirteen percent of the members of the refugee households did not eat because they lacked the financial resources to obtain enough food (Table 1). The highest and lowest average is related to question 3 (Eat a limited variety of foods) and question 9 (Go a whole day and night without eating), respectively. About 60 % of households had at least one of the different ranges of anxiety, including rarely, sometimes and often. Also, the statistics of kurtosis and skewness show that the distribution of all questions is normal.

**Fig. 4:**
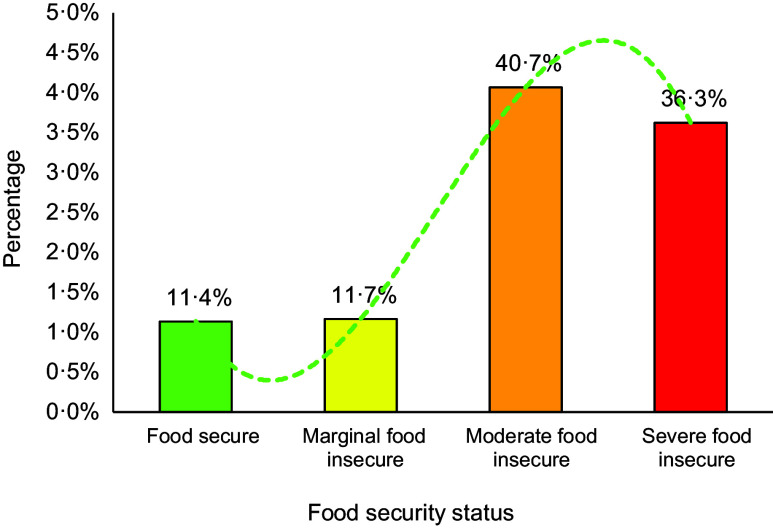
The status of Afghan refugees‘ food insecurity residing in Tehran province, Iran

Table 3 shows that only 11·3 % of the refugees were food-secure; 11·7 %, 40·7 % and 36·3 % were marginally, moderately and severely food-insecure, respectively. Of the total sample, about 88·7 % face food insecurity from the range of marginal to severe (Fig. 4). All the socio-economic factors analysed have normal distributions.

**Table 3:** Food security status of Afghan refugees in urban slums

Category	Food secure	Marginal food insecure	Moderate food insecure	Severe food insecure
Number	36	37	129	115
Percentage	11·3	11·7	40·7	36·3
Dependent variable	11·3	88·7		

Reference: authors’ results.

### The description of coefficients

The estimated multivariable regression model considering food security total score as a dependent variable is shown in Table 4. Before the estimation of the model, the multi-collinearity of all factors was tested by using variance inflation factors. All variance inflation factors were less than five, which demonstrated that the estimated model did not have multi-collinearity^(27)^. The dependent variable of the first model was the total score of HFIAS with the observed range of 1–27 in which the lower score is the better food security status vice versa.

**Table 4:** Factors associated with food insecurity score of Afghan refugees in urban slums

Factors	Coefficient	*t*	Lower bound	Upper bound	Variance inflation factor
Constant	10·632	6·564***	7·444	13·819	–
Age of HH	−0·018	−0·532	−0·084	0·048	3·194
Education level of HH	−0·071	−0·125	−1·189	1·047	1·264
Employment status of HH	0·958	2·897***	0·307	1·610	1·151
Income level	−1·438	−3·800***	−2·182	−0·693	1·542
Length of stay in Iran	0·023	0·828	−0·031	0·077	1·646
Rent amount (million Rial)	1 52 × 10^-6^	1·157	0·000	0·000	1·062
Rahn (million Rial)	0·004	0·209	−0·034	0·042	1·197
House size	−0·024	−3·028***	−0·040	−0·008	1·517
Distance to city centre	0·060	2·087***	0·003	0·117	1·231
Frequency of travel to Afghanistan	0·241	2·294***	0·034	0·448	1·260
The number of male children	1·488	2·785***	0·436	2·540	4·768
The number of female children	1·014	2·035**	0·033	1·994	4·945
The number of students	−0·095	−0·255	−0·825	0·636	2·862
The number of employed members	−0·523	−1·447	−1·234	0·188	2·308
The number of disease members	0·713	1·273	−0·389	1·814	1·140
The number of illiterate members	−0·711	−1·431	−1·688	0·267	4·828
The number of elementary members	−1·574	−2·942***	−2·626	−0·521	4·367
The number of high school members	−1·202	−2·246**	−2·256	−0·148	3·626
The number of university members	−0·002	−0·005	−1·017	1·012	1·398
Age of mother	0·088	2·786***	0·026	0·150	2·935
Smoking status of HH	−0·614	−0·990	−1·834	0·607	1·159
Personal saving in Banks	−1·276	−1·869**	−2·619	0·068	1·358

Rial is the Iranian currency. HH, Head of Household.

**,***Significant level at 5 % and 1 %.

Employment status was significantly and inversely associated with refugee households’ food insecurity (the range of employment is from permanent job to unemployed). The inverse association of income level with food insecurity suggests that higher income level of households, as expected, is associated with greater food security score. House size was inversely and significantly associated with food insecurity, while the distance from the city centre and frequency of travel to Afghanistan had a direct association with food insecurity. Regular frequent travel to Afghanistan and more distance of Afghan household’s house from the central markets was associated with greater food insecurity. A household with more male and female children was more food insecure. The number of elementary, high school and university members of Afghan refugees was significantly and inversely associated with food insecurity. The age of mother was directly associated with food insecurity. Finally, personal saving was inversely associated with food insecurity. Households with more personal savings had better food security than those with lower savings.

To investigate the third hypothesis, multivariable regression models were estimated separately for three categories of food insecurity: anxiety and uncertainties, insufficient quality and insufficient food intake. Three economic and financial factors – income level, house size and personal saving – were associated with the food insecurity of Afghan refugee households. Greater asset of households was associated with less worry about preparing members’ needed foods and less anxiety and uncertainty of inadequate monetary resources. Some factors, including employment status, income level, house size and the number of elementary members, were indirectly associated with insufficient quality, whereas the length of stay in Iran, distance from the city centre, frequency of travel to Afghanistan, the number of male children and age of mother were directly associated (Table 5). Income level, house size, the number of elementary, high school and university members were inversely associated with insufficient food intake.

**Table 5:** Factors associated with anxiety and uncertainty, insufficient quality and insufficient food intake of Afghan refugees in urban slums

Factors	Anxiety and uncertainty				Insufficient quality				Insufficient food intake			
	Coefficient	*t*	Lower bound	Upper bound	Coefficient	*t*	Lower bound	Upper bound	Coefficient	*t*	Lower bound	Upper bound
Constant	2·065	6·339***	1·023	3·825	4·978	6·002***	2·925	6·021	3·537	4·121***	2·968	4·122
Age of HH	0·001	0·036	−0·981	0·862	−0·012	−0·701	−0·983	0·821	−0·007	−0·393	−0·021	0·001
Education level of HH	−0·022	−0·196	−0·091	0·068	0·077	0·264	−0·541	0·874	−0·122	−0·405	−0·215	−0·042
Employment status of HH	0·045	0·681	−0·052	1·020	0·629	3·712***	0·021	1·021	0·325	1·854	0·251	0·524
Income level	−0·225	−2·958***	−1·015	0·025	−0·604	−3·116***	−1·125	−0·023	−0·680	−3·393***	−0·824	−0·479
Length of stay in Iran	0·004	0·681	−0·070	0·072	0·030	2·112**	−0·210	0·562	−0·008	−0·545	−0·024	0·007
Rent amount (million Rial)	1·82 × 10^−7^	0·691	0·011 × 10^−7^	2·68 × 10^−7^	8·89 × 10^−7^	1·320	7·21 × 10^−7^	9·83 × 10^-7^	5·47 × 10^−7^	0·785	4·18 × 10^−7^	6·74 × 10^−7^
Rahn (million Rial)	−0·002	−0·470	−0·060	0·012	0·000	−0·051	−0·021	0·035	0·003	0·285	0·001	0·004
House size	−0·004	−2·384**	−0·091	0·001	−0·008	−1·965**	−0·052	0·012	−0·011	−2·737***	−0·125	−0·001
Distance to city centre	0·011	1·905	−0·182	0·082	0·028	1·929**	−0·061	0·843	0·019	1·265	0·002	0·052
Frequency of travel to Afghanistan	0·021	1·015	−0·352	1·012	0·113	2·091**	−0·024	0·937	0·114	2·055**	0·012	0·210
The number of male children	0·145	1·346	0·002	0·984	0·665	2·431**	0·214	0·958	0·725	2·561**	0·682	0·815
The number of female children	0·124	1·241	0·004	0·837	0·462	1·813	0·123	0·921	0·406	1·539	0·298	0·531
The number of students	0·031	0·413	−0·002	1·012	0·040	0·213	−0·025	0·084	−0·156	−0·792	−0·285	−0·082
The number of employed members	−0·062	−0·856	−1·021	0·082	−0·131	−0·706	−0·421	−0·028	−0·311	−1·623	−0·462	−0·214
The number of disease members	−0·029	−0·259	−0·987	0·521	−0·078	−0·273	−0·214	−0·013	0·850	2·864**	0·695	1·031
The number of illiterate members	0·009	0·092	−0·521	0·086	−0·283	−1·113	−0·451	−0·053	−0·429	−1·629	−0·632	−0·351
The number of elementary members	−0·194	−1·806	−1·021	−0·001	−0·640	−2·335**	−1·021	−0·216	−0·772	−2·725***	−0·852	−0·643
The number of high school members	−0·136	−1·262	−0·982	−0·021	−0·517	−1·886	−0·751	−0·301	−0·558	−1·966**	−0·712	−0·381
The number of university members	−0·065	−0·623	−0·865	0·009	−0·076	−0·286	−0·963	−0·045	0·149	0·545	0·112	0·215
Age of mother	0·010	1·561	−0·156	0·098	0·036	2·244**	0·014	0·084	0·041	2·466**	0·012	0·061
Smoking status of HH	−0·036	−0·290	−0·451	−0·004	−0·318	−1·001	−0·541	−0·119	−0·282	−0·859	−0·381	−0·192
Personal saving in Banks	−0·424	−3·087***	−1·012	−0·012	−0·632	−1·809	−0·895	−0·428	−0·334	−0·923	−0·421	−0·281

Rial is the Iranian currency. HH, Head of Household.

**,***Significant level at 5 % and 1 %.

On the other hand, frequently travelling to Afghanistan, the number of male children, the number of disease members and the age of the mother were directly associated with the food insecurity of Afghan refugee households. Income level and house size were associated with all three categories of food insecurity.

## Discussion

Understanding Afghan refugee food insecurity status in Iran will provide the Iranian government, internal and international non-governmental organisations, civil society and global institutions with opportunities for improving refugees’ quality of life. In this study, four hypotheses were proposed. The first hypothesis was verified. Of the total sample, about 88 % of Afghan refugee households suffered from food insecurity, and about 36 % of them faced severe food insecurity. This high prevalence of food insecurity showed that the Afghan refugees do not have an appropriate dietary status. As the literature shows, the status of the food security of Iranian households is better than an Afghan household in Tehran province, the target location of this study. Mohammadi *et al.* (2013) reported that about 44 % of the Iranian population in the same location faced food insecurity^(31)^. Also, another study showed that about 48 % of Iranian households faced food insecurity in Tehran province^(30)^. Pakravan and Mohammadi-Nasrabadi (2020) reported that about 75 % of Afghan refugees faced food insecurity in Southern areas of Tehran province^(21)^. The comparison between the general population and Afghan refugees showed that Afghan households have low levels of food security and need urgent intervention.

To check the second hypothesis, we found that household characteristics were associated with Afghan refugees’ category of food security. Based on the descriptive analysis and multivariable regression model, a seasonal or permanent job of head of the household was associated with a better food security status of Afghan refugee households. This indirect association may be related to the potential of the heads for gaining enough money to prepare the needed foods for their family^(22)^. The probability of food insecurity was higher^(32)^ in a household whose head is unemployed. This result may be related to physical health and poor mental status via paths that include income deficiency, reduction of physical and mental activity, loss of status and self-esteem and unhealthy behaviours. Some authors argue that unemployment is also directly associated with food insecurity, due to the exacerbation of occupation search strategies and the workload of the household^(32)^. The size of the house or apartment was also indirectly associated with food insecurity. The measurement of the house or apartment’s area is a proxy for wealth^(21,23,30)^. Some researchers found a similar association of house or apartment size with food security^(33,34)^. Thus, households with higher wealth and assets can easily spend more income on food and have higher food security than their lower-income counterparts.

Distance from the city centre was indirectly associated with the food insecurity of Afghan refugee households. Many poor communities are located a substantial distance from supermarkets and effectively live in a ‘food desert,’ an inhabited area with negligible access to nutritious and healthy food because of geographical position, in-store choice or affordability^(35,36)^. They are not able to prepare the needed foods whenever they want and need, because these markets are not frequently available^(37)^. Therefore, they have to pay more to access these markets^(37)^.

There was a significant direct association between frequent travel to Afghanistan at regular intervals and Afghan refugees’ food insecurity. This finding is consistent with the results of other studies^(8,38,39)^. There are several explanations for this finding: First, refugees who visit their country and pay money to travel must dedicate a portion of their income to these trips, so some may need to reduce their food and non-food expenditures to fund these travels. Second, when refugees return from Afghanistan, it may take them a long time to reintegrate into Iranian communities. For example, it may be difficult to find stable employment and affordable, healthy food. Third, based on our face-to-face interviews, Afghan refugees who return to their country are more likely to have a household confronting arduous financial obstacles in Afghanistan. As a result, these refugees may assign a portion of their income to help the kindred who is still in Afghanistan. Frequent travels to Afghanistan may make it harder to find a permanent job in Iran. Earlier studies with similar findings^(8,38,39)^ have proffered similar explanations for the food insecurity of refugees who frequently travel to their home countries. When they fail to resettle in a host country for a long period, refugees continue to cope with the loss of their culture, country, profession, language, friends, family and future plans^(8)^ To summarise, all of these reasons may lead to being food insecure. Increasing the number of male and female children was directly associated with Afghan households’ food insecurity. Refugees who have more children are forced to try harder to provide sustainable resources for themselves. In these cases, extra income is needed from the household head or other adults in the household. Due to many restrictions on refugees in Iran, they are not hired for jobs that reflect their skill levels, and most works are in lower-income positions. Household expenditures are increasing much more rapidly than worker wages. As Iran suffers from a high level of unemployment, refugee youngsters have difficulty finding a job. These difficulties have negative consequences on accessibility, and eventually, the food insecurity of refugees who have more male and female children^(40)^. Also, the association of number of male children is more strongly related to food insecurity than number of female children, because physiologically male children consume more food compared to female children^(30)^. An increase in the number of elementary and secondary members of a household was associated with a lower food insecurity score.

Enhancing the level of education of family members helps them to become familiar with different aspects of dietary diversity and the importance of having an appropriate foodstuff combination. Also, some researchers believed that education level harms food insecurity; others argued that as education costs are high (including providing own uniforms, books and materials), each additional student within a household was indirectly associated with households’ food security due to decrease in the available money for foods^(41,42)^. There is not the same obstacle in Iran because education is free at elementary and secondary education levels.

Finally, personal saving was inversely associated with the food insecurity of Afghan refugee households. This result may be related to the decrease in anxiety and the stress of providing financial resources for the needed foods. They can access this saving whenever they want and need it. Additionally, the role of personal saving for the reduction of anxiety and stress, which is the third hypothesis, is entirely evident.

The results confirmed the third hypothesis. About 60 % of Afghan households experienced at least one of the different degrees of anxiety. An extensive study of mental health of refugees argued that the prevalence of mental disorders is in the range of 30–40 % among refugees, and about 55 % of refugees had at least one psychiatric disorder^(43)^. Economic and financial assets, including income level, house size and personal saving of Afghan refugee households were inversely associated with anxiety and uncertainty. The first question of the HFIAS questionnaire, which is used to assess the anxiety of a household, is directly related to the potential of the household to acquire foods during the previous month. Therefore, a household’s economic and financial assets, which assess accessibility as a proxy of food security, is an indispensable factor in reducing the stress and anxiety of the households^(26,37)^. In the context of the acculturation process, poor economic conditions and an unclear residence permission status are considered as a stressor for refugees and immigrants^(44)^. Many studies confirm the importance of post-migration status for food security status. Steel *et al*. (2002) claimed that several post-migrations, socio-economic determinants are not associated with mental illness among Vietnamese refugees^(45)^. Some authors argued that various socio-economic determinants are associated with the anxiety and stress, as mental disorders, in the Middle East^(46–48)^. Finally, the association of socio-economic factors was different for anxiety, insufficient food quality and insufficient food intake. Therefore, the fourth hypothesis was verified by these results. The income level has a similar association with all the dependent variables, including insufficient food intake, insufficient quality and anxiety. House size was indirectly associated with anxiety, insufficient food quality and insufficient food intake.

### Limitations

This study has several limitations. First, some Afghan refugees did not trust our interviewers because some of them were illegal and undocumented refugees and were fearful and cautious in answering interviewers’ queries. About 15 % of the selected refugees refused an interview, and we had to substitute a new sampling. We explained that the results would be helpful for their resettlement and for reducing their problems and that the data would be confidential and used in academic research. Second, because of the cross-sectional design, this study does not allow to draw causality. Third, we could not easily find Afghan refugees in specified locations due to the scattered population across the province, and data collection was expensive, complicated and time-consuming. To reduce the time of the study, we recruited several interviewers. Fourth, it was difficult to distinguish between refugees who entered Iran voluntarily and those who had fled to Iran due to fear of the arduous situations and the many restrictions in Afghanistan such as race, religion, nationality and political opinions. We asked them whether they had a passport or not to distinguish these two groups. Finally, distinguishing between Afghan people who were born in Iran and those who were refugees was difficult. To do so, we asked them whether they had an Iranian birth certificate.

## Conclusion

A high proportion of Afghan refugees live under severe food insecurity conditions in Tehran province in Iran. To improve food security, interested and related organisations and institutions must develop bi- and multi-lateral collaborations. About three-quarters (77 %) of Afghan refugees need the immediate intervention of the local government or related institutions like the Ministry of Refugees and Repatriations of Afghanistan or the United Nations High Commissioner for Refugees. To improve the food security status of Afghan refugees, determining socio-economic factors play a crucial role because the low-income governments can use policies other than income policy based on the association of these factors with food insecurity. Although the employment of the head of refugee households was directly associated with food security, some Iranians, as in other nations, may believe that Afghan refugees will take the resources of the host country and fill their job positions as intruders. Local non-governmental organisations can conciliate these beliefs and lead to a better understanding of cultures and help refugees find a job without any intellectual engagement among Iranian people. Economic factors, including personal saving, house size and households’ income, were critical factors in decreasing the anxiety and uncertainty of Afghan refugees’ food security. Financial and monetary networks in Iran, and specifically, the Ministry of Economic Affairs and Finance and the Central Bank of Iran, should provide simple access to financial supports like a long-term loan, self-employment packages and opening a bank account for Afghan refugees, benefitting both Iranians and refugees.

## Supplementary material

For supplementary material accompanying this paper visit https://doi.org/10.1017/S1368980021004043. click here to view supplementary material
