# Supplementary material for: The assessment of an extended set of socio-economic determinants to explain anxiety and uncertainty, insufficient quality and food intake of Afghan refugees
Source: Public Health Nutr. 2021 Sep 23;25(3):554–64. doi: 10.1017/S1368980021004043 (PMC9991560; doi:10.1017/S1368980021004043)
Supplement: Supplementary file 1 [file S1368980021004043sup001.docx]

***Appendix***

***Table A1- The description of variables in the multivariable regression model***

| **Factors** | **Scale** | **Definition** |
| --- | --- | --- |
| Food security score | (0-27) | 0= food secure status; 27=severe food insecure status |
| *Anxiety and uncertainty* score | (1-3) | 1= low anxiety; 3= high anxiety |
| *Insufficient quality score* | (1-9) | 1= low insufficient quality; 9= high insufficient quality |
| *Insufficient food intake* score | (1-15) | 1= low insufficient food intake; 15= high insufficient food intake |
| Age of HH | Year | The age of head of Afghan households |
| Education level of HH | (1-4) | 1=Illiterate level; 2=Rudimentary level; 3=High school; 4= University level |
| Employment status of HH | (1-3) | The situation of head’s employment Group1=Permanent, Group2=Seasonal, Group 3=unemployment; the base group is the third group=0) |
| Income level | (1-4) | Level 1=million Rial (1=<45$), Level 2=million Rial (2=), Level 3=million Rial (3=); Level 4=(4=) million Rial. |
| Length of stay in Iran | Year | The number of years the household members have lived in Iran |
| Rent amount (million Rial) | Rial | The amount of rent of house |
| Rahn (million Rial) | Rial | The amount of deposited money in refundable-deposit-lease-contract (million Rial/year). (This is the refundable money as a guaranty which renter will give back home absolutely flawless and pay the monthly rent on time. After finishing the time of contract, he/she will retake this deposited money- This type of contract for renting a home is known “Rahn” in Iran). |
| House size | M^2^ | The size of Afghan refugees’ house (m2) |
| Distance to city center | Kilometer | The distance of the refugees’ dwelling to the city center (in kilometers) |
| Frequency of travel to Afghanistan | Times | The frequency of visits to Afghanistan |
| Male children | Number | The number of male children of Afghan household |
| Female children | Number | The number of female children of Afghan household |
| Students | Number | The number of students within a household |
| Employed members | Number | The number of employed members within a household |
| Disease members | Number | The number of disease members within a household |
| Illiterate members | Number | The number of illiterate members within a household |
| Elementary members | Number | The number of elementary members within a household |
| High school members | Number | The number of high school members within a household |
| University members | Number | The number of university members within a household |
| Age of mother | Year | The age of mother of Afghan household |
| Smoking status of HH | Number | The number of used cigarette during a day |
| Personal saving in Banks | (1-4) | 1= Less than 500 million Rial; 2= Between 500 and 1000 million Rial; 3= Between 1000 and 5000 million Rila; 4= More than 5000 million Rial |

**Note: HH is Head of Household; Rial is the Iranian currency**
